# How university physical education instructors’ political literacy shapes student values: the mediating role of teacher educative efficacy in the Chinese context

**DOI:** 10.3389/fpsyg.2026.1782929

**Published:** 2026-04-02

**Authors:** Xiao Qiaoli, Liu Bing

**Affiliations:** 1 Physical Education College, Shanghai University, Shanghai, China; 2 Sports Science Research Center, Shanghai University, Shanghai, China

**Keywords:** mediation analysis, physical education, political literacy, structural equation modeling, teacher educative efficacy, university PE instructors, value-based education

## Abstract

**Background:**

In the context of China’s comprehensive implementation of the fundamental task of “Fostering Virtue through Education,” integrating values education into university physical education (PE) and elevating it from “skill delivery” to “character shaping” has become a central educational issue of China. However, robust empirical evidence on how PE instructors’ political literacy drives this process remains scarce.

**Methods:**

This study draws on survey data from PE instructors across 20 Chinese provinces and municipalities, collected from December 2025 to January 2026. Using structural equation modeling and mediation analysis, it systematically examines the internal mechanism through which teachers’ political literacy influences student values development.

**Results:**

(1) University PE instructors’ political literacy is a multidimensional construct comprising political understanding, identification, faith, and practice; (2) teachers’ “educative efficacy” plays a substantially mediating role between political literacy and effectiveness in fostering student values; and (3) three “transmission delays” exist within this pathway: the delay in translating political cognition into teaching practice, the delay in sublimating classroom fun into value internalization, and the delay in converting educative efficacy into educational outcomes.

**Conclusion:**

The accumulation of political literacy is driven by the endogenous motivation of self-efficacy, its deepening relies on external support from collaborative platforms, and its sustained development depends on the iterative momentum provided by feedback mechanisms. Consequently, this study constructs an integrated enhancement path synergizing “endogenous motivation stimulation, external environment optimization, and developmental mechanism guarantee.” This research elucidates the universal mechanism of how teachers translate personal belief systems into effective teaching practices, providing practical guidance for the professional development of PE instructors in the new era.

## 1 Introduction

Globally, the integration of values education into disciplinary teaching, marking a paradigm shift from the delivery of knowledge and skills to the holistic development of the person, has become a significant trend (Lovat, 2011; Griban et al., 2022). In China, this paradigm is concretely realized through the “Curricular Ideological and Political Education” reform under the fundamental task of “Fostering Virtue through Education”. This reform mandates the systematic integration of ideological and political education into all curricula, including university PE (Wang and Zhao, 2024; Liu P. et al., 2021; Liu X. et al., 2021). However, transforming university PE, traditionally centered on skill instruction, into an educational front that consciously shapes student values presents profound practical challenges. Central to this challenge is the teacher’s capacity to effectively translate political and ideological concepts into meaningful teaching practices. Internationally, a substantial body of literature has examined how teachers’ internalized belief systems shape their pedagogical practices and, consequently, student development. Concepts such as “teacher beliefs” (Goddard et al., 2015), “moral education competence” (Tao et al., 2025), and “teacher competence” (Blömeke et al., 2022) all point to a common theoretical insight: effective values education requires teachers to possess not only subject knowledge but also the capacity to translate societal values into pedagogically meaningful practices. Within the Chinese context, this capacity is conceptualized as teachers’ “political literacy,” a construct that, while linguistically and culturally situated in China’s ideological education discourse, resonates with these international concepts.

Existing literature has begun to explore the teacher competencies required for this integrated teaching model. Prior research has often focused either on foundational personal virtues (Angelini et al., 2025; Bobkova et al., 2020) or on the application of political knowledge (Zibenberg and Grinshtain, 2024; Ramírez-Biondolillo, 2025). We contend that effective values education in a specialized discipline like PE necessitates a more complex and practice-oriented professional construct. Therefore, we define the political literacy of university PE instructors as a multi-dimensional professional competency encompassing systematic political understanding, profound political identification, steadfast political faith, and, most critically, the ability to organically integrate these elements into professional teaching practice—political practice. It is precisely this integrative capability that positions it as a key variable determining the effectiveness of values education in PE.

Within the Chinese context, deficiencies in teachers’ political literacy manifest primarily in two practical dilemmas. First, there is a widespread issue of superficial integration: teachers with an insufficient theoretical foundation struggle to establish deep connections between socialist core values and the spirit of sportsmanship, resulting in a “tagged-on” and “mechanical” approach to values education (Shang, 2022; Huang et al., 2025). Second, and more fundamentally, there is the phenomenon of “value transmission attenuation” (Dong et al., 2024). Although sports activities inherently provide scenarios for cultivating collectivism, rule consciousness, and other values, their highly practical nature often confines education to the behavioral level. Consequently, students may fail to internalize experiences, such as teamwork, into deeper value convictions, such as a sense of national identity and sentiment (Liu and Xiao, 2024).

Crucially, a significant research gap exists regarding the internal mechanism through which political literacy ultimately influences student values. While its importance is acknowledged, the internal psychological processes and behavioral pathways linking a teacher’s literacy to student value internalization remain a “theoretical black box” (Mingxu et al., 2025). Research must move beyond simple correlations and construct dynamic models, such as “political literacy → teaching process → value shaping,” to unveil this underlying mechanism.

To address this research gap, the present study draws on social cognitive theory (Bandura, 1997) and introduces teacher educative efficacy as a core mediating variable. Bandura defined self-efficacy as “an individual’s belief in their ability to organize and execute courses of action required to achieve specific attainments.” In the context of this study, teacher educative efficacy refers to PE instructors’ belief judgments about whether they can successfully accomplish the tasks of values education and effectively promote students’ values development. This sense of efficacy stems not only from teachers’ evaluation of their own teaching skills but also from their confidence in translating political literacy into specific teaching strategies (Pereira et al., 2020). Therefore, teacher educative efficacy connects teachers’ intrinsic belief systems with their explicit teaching behaviors and serves as the key psychological mechanism explaining how political literacy translates into educational effectiveness (Shang et al., 2022).

Based on a national survey of Chinese university PE instructors and utilizing structural equation modeling, this research seeks to: (1) validate the multidimensional structure of PE instructors’ political literacy; (2) test the core hypothesis that teacher educative efficacy plays a significant mediating role between political literacy and effectiveness in fostering student values; and (3) identify potential “transmission delays” within this pathway, thereby providing a nuanced explanation for the observed “attenuation” problem. Through the aforementioned work, this study aims to unpack the construct of political literacy, identify its generalizable psychological components, and thereby contribute theoretical insights and an empirical model to the universal process of how teachers translate their personal belief systems into effective values education practices.

## 2 Literature review and theoretical model

### 2.1 Literature review

#### 2.1.1 International perspectives on values education in PE and the absence of the teacher’s role

In China, the value education within the PE curriculum shares similar educational objectives with “value education,” “moral education,” and “civic education” in subject teaching globally—both aim to foster students’ holistic development and cultivate positive civic character (Yang et al., 2025). However, international comparative research reveals that while sports, as a sociocultural practice, universally serve a value-shaping function across cultures, the pathways to achieving this are deeply embedded within specific sociocultural contexts (Weinberg et al., 2019). For instance, in the United States, democratic values are often concretized through leadership training and the principle of fair competition in sports (McKown et al., 2025). Germany systematically integrates self-management and disciplinary norms into team sports like football to convey its professional ethics (Grossmann and Lames, 2015). In China, PE possesses distinctive Chinese characteristics. It is not merely a general form of character education but is explicitly centered on cultivating and practicing the Core Socialist Values, emphasizing the socialist orientation of education. Compared to the more commonly discussed universal democratic values or critical citizenship education in the West, Chinese PE places greater emphasis on fostering collectivism, national identity, and social responsibility (Li, 2023).

However, existing research generally exhibits a tendency to “prioritize pathway design over the practitioner.” Most literature focuses on structural factors like curriculum design and teaching models, while relatively neglecting the central role of teachers as the ultimate agents of value transmission (Cheng and Ji, 2025). PE teachers should not be passive conduits of values but active agents who filter, interpret, and contextualize educational content (MacLean et al., 2015). Particularly for the Chinese “Curricular Ideological and Political Education” paradigm, which demands highly conscious integration, the teachers’ own political literacy—their ability to understand, identify with, and creatively translate political concepts—becomes a decisive factor for its success. In-depth exploration of this critical variable remains insufficient in current academic discourse.

#### 2.1.2 The conceptual evolution of teachers’ political literacy and research limitations

In international academia, the ways in which teachers’ internalized value systems shape student development through educational processes have long been a focal point of multidisciplinary inquiry. Relevant research has developed along multiple trajectories. One line of inquiry conceptualizes teacher beliefs as tacit assumptions about knowledge, learning, and society that fundamentally shape pedagogical decisions (Pajares, 1992). Another strand examines teachers’ role as civic educators, emphasizing their understanding of democratic processes and their capacity to foster students’ civic engagement (Westheimer, 2022). A third body of work focuses on teachers’ moral reasoning competence, highlighting their ability to navigate ethical dilemmas and facilitate students’ moral development (Wainwright and Lind, 2017). More recently, the notion of teachers as change agents has further foregrounded teachers’ potential to transmit, interpret, and occasionally critique societal values within classroom contexts (van der Heijden et al., 2015). Although these research trajectories differ in their conceptual frameworks and value orientations, they collectively converge on a core proposition: how do teachers’ internalized value systems translate into educational practices that shape students’ civic and moral development?

Within the Chinese context, this proposition has been theorized and operationalized through the indigenous concept of political literacy. Its conceptual evolution exhibits a clear historical trajectory: early research focused primarily on teachers’ political stance and ideological beliefs (Mirra and Morrell, 2011); mid-stage research expanded to encompass political acumen and the capacity for value-based guidance (Geller, 2020); and recent scholarship has gradually developed a systematic tripartite framework of “value core—cognitive structure—practical ability,” emphasizing the integrative role of political identification, affect, and belief in teaching and curriculum (Lu and Lin, 2025). Whereas international constructs tend to be grounded in discourses of democratic citizenship education and individual moral development, the Chinese concept of “political literacy” is deeply embedded in the policy context of “Curriculum-based Ideological and Political Education” and the fundamental task of “Fostering Virtue through Education,” reflecting a distinct institutional orientation and value-integration imperative. Yet, despite these discursive differences, the fundamental question it addresses—how teachers intentionally and effectively shape students’ civic and moral development through their internalized value systems—resonates deeply with the core concerns of international scholarship, thereby laying a theoretical foundation for cross-cultural dialogue and conceptual integration.

Despite this conceptual enrichment, existing research still suffers from a dual limitation. On a theoretical level, most discussions remain centered on the general teacher population, failing to adequately reveal the specific expression mechanisms of political literacy within the particular subject field of PE—for instance, how political concepts are translated into physical practice and team interactions (Cheng and Ji, 2025). On an empirical level, a significant “mechanism black box” exists. There is a severe lack of empirical testing of the transmission chain detailing “how political literacy influences student values through specific teaching processes” (Ma and Yang, 2019). This disconnection between theoretical depth and empirical evidence directly contributes to the practical dilemma of “ideological and political elements being mechanically piled on” while “endogenous motivation for value formation remains insufficient” (Ma and Yang, 2019). Consequently, constructing a theoretical model grounded in the distinctive characteristics of sports pedagogy and capable of revealing the underlying influence mechanism is particularly urgent.

### 2.2 Theoretical model and research hypotheses

#### 2.2.1 Theoretical foundation

The theoretical foundation of this study is rooted in the Marxist theory of the all-round development of the individual, which emphasizes the active role of education in shaping the human being as the “totality of social relations” (Peretti and Ribeiro, 2025). Within this framework, university PE instructors transcend the traditional role of “skills coaches” to become guides in the formation of students’ values. Within China’s “Big Ideological and Political Education” landscape, teachers’ political literacy constitutes the key professional support for fulfilling their educational responsibility. It enables teachers to: methodologically, move beyond superficial skills and deeply excavate the opportunities for values education inherent in the collective nature, competitiveness, and rule-based structure of sports; in practice, construct an educational network that synergizes teaching, discipline, and daily life; and through their own demonstrative effect, provide students with a perceptible and emulable behavioral model.

#### 2.2.2 Model construction: introducing educative efficacy as the core mediator

To explore the influence mechanism from teachers’ “political literacy” to students’ “values effectiveness,” this study introduces teachers’ educative efficacy as a core mediating variable, whose theoretical foundation is directly derived from Bandura’s social cognitive theory, particularly the core construct of “self-efficacy” (Bandura, 1997). Bandura defined self-efficacy as “an individual’s belief in their ability to organize and execute courses of action required to achieve specific attainments.” In the context of this study, we specify it as PE instructors’ belief judgments about whether they can successfully accomplish the tasks of “Curriculum-based Ideological and Political Education” and effectively promote students’ values development. This sense of efficacy stems not only from teachers’ evaluation of their own teaching skills but also from their confidence in translating political literacy into specific teaching strategies (Pereira et al., 2020). Therefore, “educative efficacy” represents the concretization and application of self-efficacy in the specific professional practice of values education. It connects teachers’ intrinsic belief systems with their explicit teaching behaviors and serves as the key psychological mechanism explaining how “political literacy” is translated into educational effectiveness (Shang et al., 2022).

**Figure 1:**
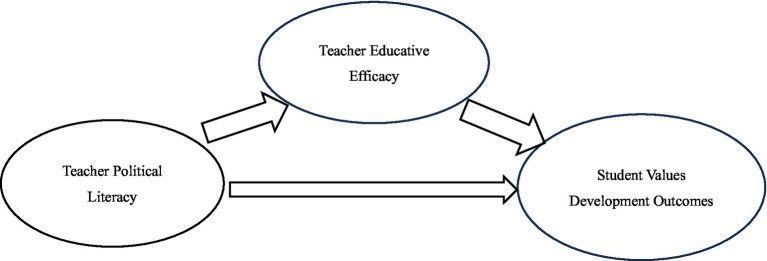
The mediating model of university PE instructors’ educative efficacy between political literacy and the student values development outcomes.

Based on this reasoning, this study constructs a mediation model (shown in Figure 1): Teacher Political Literacy (independent variable) influences Student Values Development Outcomes (dependent variable) through Teacher Educative Efficacy (mediating variable). This model aims to unveil the “literacy → belief → outcome” transmission mechanism, providing a systematic theoretical framework to explain the relationships between the core variables.

### 2.3 Research hypotheses

Based on the theoretical model outlined above, this study proposes the following hypotheses:

*H1*: University PE instructors’ political literacy has a significant positive impact on their effectiveness in fostering student values.

*Elaboration*: The depth of a teacher’s understanding of political theory, the strength of their identification with core values, and their practical ability to organically integrate ideological and political elements into sports instruction collectively form the foundation for influencing student values through PE.

*H2*: Teacher educative efficacy plays a significant mediating role in the relationship between political literacy and effectiveness in fostering student values.

*Elaboration*: Teachers with high political literacy are more likely to develop strong educative efficacy—a firm belief in their capability to successfully accomplish values education tasks. This efficacy belief drives them to more proactively refine teaching strategies, engage more persistently in teacher-student interactions, and respond more effectively to pedagogical challenges, thereby ultimately translating their political literacy more fully into tangible student values development outcomes.

## 3 Methodology

### 3.1 Participants

The participants of this study were physical education (PE) teachers from various universities across China. A total of 207 questionnaires were distributed and recovered, resulting in an effective response rate of 100%. To ensure the representativeness of the sample, the respondents were recruited from 20 provinces and municipalities, covering the Eastern, Central, and Western regions of China, such as Guangdong, Hunan, and Shaanxi. The demographic characteristics of the participants are summarized in Table 1.

**Table 1:** Demographics of the participants (*N* = 207).

Variable	Category	Frequency (*n*)	Percentage (%)
Gender	Male/female	128/79	61.8/38.2
Teaching experience	<5 years/5–10 years/11–20 years/>21 years	44/31/38/94	21.3/15.0/18.4/45.4
Professional title	Assistant/lecturer/associate prof./prof.	32/72/79/24	15.5/34.8/38.2/11.6
Region	Eastern/central/western	104/62/41	50.2/30.0/19.8
University type	Teachers from key universities/teachers from general universities	75/132	36.2/63.8

### 3.2 Instruments

#### 3.2.1 Teacher political literacy scale

This study defines teacher political literacy as a multidimensional construct, referring to the professional competency of teachers to systematically understand, internally identify with, firmly believe in political ideas, and creatively translate them into PE teaching practices. Drawing on the communication effects theory from communication studies (Moreno-Casado et al., 2023), we conceptualize teachers as “encoders” and students as “decoders.” Teachers’ political literacy ultimately influences students’ emotions, attitudes, and behaviors through the communicative process of teaching interactions over time.

Integrating the essential “body–mind unity” attribute of the sports discipline, we constructed four dimensions of political literacy: Political Understanding refers to teachers’ accurate interpretation ability regarding national strategies and sports policies (e.g., “Sports Power,” “Integration of Sports and Education”) and their cognitive level of the specific principles of ideological and political education in PE curricula (MacLean et al., 2015). Political Identification refers to teachers’ embodied, emotional identification with Core Socialist Values, grounded in the inherent ethos of sports, such as collectivism and fair competition (Wang, 2013). Political Faith refers to teachers sublimating the patriotic tradition and striving spirit inherent in sports into a firm educational belief and political sense of mission, encapsulated as “fostering virtue through sports, serving the nation through sports.” Political Practice refers to teachers’ instructional design and implementation strategies for organically transforming ideological elements into embodied learning experiences within motor skill teaching. These four dimensions form a dynamic cycle: understanding is the foundation; identification deepens understanding and sublimates into faith; faith ultimately drives practice; and practice, in turn, tests and deepens understanding. The final scale comprises 4 first-level dimensions, 11 s-level dimensions, and 39 items. Reliability analysis indicated that the Cronbach’s *α* coefficients for the dimensions ranged between 0.933 and 0.981, demonstrating excellent internal consistency.

#### 3.2.2 Student values development outcomes scale

The operational definition of student values development outcomes is closely aligned with the fundamental objective of “Fostering Virtue through Education” in Chinese school PE for the new era. Its theoretical basis derives from national top-level design, such as the principle proposed by Chinese leadership that school sports should enable students to “enjoy fun, enhance physical fitness, develop well-rounded personalities, and strengthen their willpower,” and relevant policy documents (e.g., “Opinions on Comprehensively Strengthening and Improving School Sports Work in the New Era”) (Xi, 2022). It also incorporates academic discourse on the unique role of sports in shaping worldview, outlook on life, and values (Moravecz et al., 2025).

Accordingly, we divided this construct into four dimensions: Enjoyment Experience refers to the pleasure and enthusiasm students gain from sports participation, thereby stimulating intrinsic motivation. Physical Fitness refers to the improvement of students’ physical health and the development of good exercise habits. Character Development refers to students’ social development in areas such as integrity, cooperation, responsibility, and collective consciousness, cultivated through the spirit of sports. Willpower refers to the resilience, sense of purpose, and ability to withstand setbacks honed by students through sports activities. The final scale consists of 4 first-level dimensions, 12 s-level dimensions, and 37 items. The Cronbach’s *α* coefficients for the dimensions ranged from 0.920 to 0.971, indicating good reliability.

#### 3.2.3 Teacher educative efficacy scale

In this study, teacher educative efficacy refers to teachers’ judgment of their confidence in effectively fulfilling their values education responsibilities. It originates not only from individual self-efficacy but is also significantly influenced by collaborative support and feedback-reflection systems (Zhang et al., 2025). Its measurement encompasses three dimensions: Self-Efficacy refers to teachers’ confidence in their own educational capabilities and teaching style. Collaboration and Platform Support denotes the level of support teachers receive from teamwork and organizational culture. Feedback and Reflection indicates teachers’ initiative in pursuing professional improvement based on student outcomes and self-assessment. The finalized scale comprises 3 first-level dimensions, 7 s-level dimensions, and corresponding items. Reliability tests showed that the Cronbach’s α coefficients for the dimensions were 0.892, 0.800, and 0.922, respectively, all reaching acceptable levels.

#### 3.2.4 Validity analysis

The validity of the measurement instruments was systematically evaluated through content validity and construct validity (including structural and convergent validity).

First, content validity was established through a rigorous qualitative and quantitative development process. Following policy text analysis and an extensive literature review, the Delphi method was employed, involving a panel of 12 experts (5 from sports science, 4 from ideological and political education, and 3 from educational psychology). These experts evaluated the items based on three criteria: relevance, clarity, and theoretical representativeness, using a 4-point Likert scale (1 = “not relevant” to 4 = “highly relevant”). The Content Validity Index (CVI) was then calculated to quantify the consensus. Adhering to established methodological standards, items with an Item-level CVI (I-CVI) below 0.78 were revised or deleted. The final Scale-level CVI (S-CVI/Ave) for each scale exceeded 0.90, indicating excellent content validity.

Second, construct validity was assessed using both exploratory factor analysis (EFA) and confirmatory factor analysis (CFA). The preliminary EFA showed a Kaiser-Meyer-Olkin (KMO) value of 0.967, and Bartlett’s test of sphericity was highly significant (*χ*^2^ = 15520.423, df = 1,176, *p* < 0.001), confirming that the empirical data were exceptionally suitable for factor extraction. Subsequent CFA results indicated a satisfactory model fit for the multi-dimensional structure: CMIN/DF = 2.326, CFI = 0.910, TLI = 0.901, and RMSEA = 0.080. These indices meet the recommended academic thresholds, supporting the structural validity of the instruments.

Finally, convergent validity was verified through the calculation of Composite Reliability (CR) and Average Variance Extracted (AVE). The factor loadings for most items exceeded 0.60, and the CR values for all constructs were above 0.80, with AVE values generally meeting or approaching the 0.50 benchmark. Although high inter-correlations were observed between certain dimensions—reflecting the inherent conceptual synergy and theoretical integration of political literacy and value-based education within the Chinese “Curricular Ideological and Political Education” framework—the overall validity evidence provides a robust foundation for the subsequent structural path analysis.

To minimize the potential for common method bias (CMB) inherent in self-reported, cross-sectional data, this study implemented several procedural remedies during the data collection phase (Podsakoff et al., 2003). These included ensuring strict respondent anonymity, emphasizing that there were no right or wrong answers to reduce social desirability, and refining the item wording to ensure clarity and neutrality. Preliminary diagnostic analysis of the measurement model structure indicated that no single factor emerged as dominant, nor did a general factor account for the majority of the covariance among the measures. These procedural and structural indicators suggest that common method bias does not significantly threaten the validity of the findings.

### 3.3 Data collection

The survey was conducted from December 2025 to January 2026. Before administration, the research purpose, questionnaire structure, and confidentiality agreements were thoroughly explained to all participants to ensure the authenticity and validity of the responses. Ethical approval for the study was obtained from the Ethics Committee of Shanghai University, and all participants provided written informed consent.

### 3.4 Data analysis

This study utilized SPSS 26.0 and AMOS 24.0 for collaborative data analysis, following these specific steps:

1. *Descriptive statistics and correlation analysis*. SPSS was used to perform descriptive statistics on the sample’s demographic characteristics and core variables. Pearson correlation analysis was employed to preliminarily examine the bivariate relationships between variables.
2. *Measurement model test*. AMOS was used to conduct confirmatory factor analysis (CFA) to assess the fit of the measurement model, including factor loadings, composite reliability, convergent validity, and discriminant validity. This ensured the accuracy and validity of the construct measurements.
3. *Structural model and hypothesis testing*. After validating the measurement model, a structural equation model (SEM) was constructed to directly test the theoretical pathway “political literacy → educative efficacy → values development outcomes” proposed in this study. The overall model fit was evaluated using model fit indices.
4. *Mediation effect analysis*. To test the mediating role of educative efficacy, this study employed the Bootstrap sampling method (with 5,000 resamples) to calculate the significance of the indirect effects. By calculating the direct, indirect, and total effects, the internal transmission mechanism through which teachers’ political literacy influences student values was systematically analyzed.

## 4 Results and discussion

### 4.1 Descriptive statistics and current situation analysis

Based on the survey data, this study first conducted a descriptive analysis of the current state of the core variables. The results indicate that university PE instructors exhibit generally positive but internally uneven characteristics regarding political literacy, educative efficacy, and effectiveness in fostering student values. This is specifically manifested as a sharp contrast between “theoretical cognition being stronger than practical translation” and “individual efficacy being stronger than systemic support.”

First, a “cognition-practice gap” exists in teachers’ political literacy. The data reflect that teachers have a solid foundation at the levels of political understanding, identification, and faith: the vast majority of teachers acknowledge the necessity of integrating ideological and political theory into teaching (85.5%) and can grasp teaching direction by following political news (86.5%) and policy trends (87.4%). In terms of political identification, nearly 90% of teachers (88%) emphasize the moral education function of sports, highly identify with national strategies like the “Chinese Dream” (85.1%), and recognize the value of synergizing with curricular ideological and political education (88.9%). Political faith is also very firm, with over 90% of teachers viewing the transmission of positive values as their mission (90.3%) and valuing the inheritance of sports culture (89.4%). However, in contrast, their political practice ability is relatively insufficient: only 72.5% of teachers self-rated their practical ability as competent, and the proportion of teachers who can naturally integrate ideological and political elements into their teaching drops further to 63.8%. This gap highlights the challenge of translating internal political cognition into effective teaching behaviors.

Second, the effectiveness of values development through PE is significant, showing a positive trend of synergistic development across multiple dimensions. At the level of enjoyment experience, teachers generally observe an increase in student participation (87.9%) and actively stimulate interest through differentiated instruction (91.3%) and enriching extracurricular activities (77.8%). Regarding physical fitness, the vast majority of teachers (86.9%) acknowledge the role of sports in promoting health and observe improvements in students’ athletic performance (88.4%). In shaping character development, rule awareness (93.2%) and fostering an optimistic mindset (94.2%) are focal points. In terms of tempering willpower, resilience training (91.8%) and encouraging innovative exploration (88.9%) are widely adopted. These data collectively demonstrate that university PE courses are effective in promoting students’ holistic development.

Finally, teacher educative efficacy exhibits a dual characteristic of “strong individual capability but weak systemic support.” At the individual level, teachers’ self-efficacy is very prominent, with over 92% expressing strong confidence in their teaching (92.3%) and actively innovating teaching methods (93.2%). Simultaneously, they also demonstrate a strong sense of collaboration (86% recognize the value of cooperation) and habits of feedback and reflection (over 91% value student feedback and enhance their abilities through reflection). However, at the systemic support level, only 72.5% of teachers believe they receive adequate teaching resources and organizational support. This tension between the individual and the system indicates that the continuous improvement of teachers’ educative efficacy depends on the enhancement of external institutional guarantees and platform support.

### 4.2 Correlation analysis results

To preliminarily examine the relationships between variables, this study calculated Pearson correlation coefficients between the dimensions of the core variables (see Table 2). The results revealed significant correlation patterns within and between the variables, providing a foundation for subsequent model testing.

**Table 2:** Pearson correlation coefficients.

Variables	Political understanding	Political identification	Political faith	Political practice	Enjoyment experience	Physical fitness	Character development	Willpower	Self-efficacy	Collaboration and platform
Political identification	0.972^***^									
Political faith	0.952^***^	0.988^***^								
Political practice	0.718^***^	0.696^***^	0.709^***^							
Enjoyment experience	0.616^***^	0.621^***^	0.616^***^	0.820^***^						
Physical fitness	0.590^***^	0.591^***^	0.587^***^	0.736^***^	0.958^***^					
Character development	0.684^***^	0.705^***^	0.699^***^	0.841^***^	0.919^***^	0.877^***^				
Willpower	0.676^***^	0.686^***^	0.678^***^	0.825^***^	0.895^***^	0.845^***^	0.972^***^			
Self-efficacy	0.668^***^	0.669^***^	0.670^***^	0.855^***^	0.931^***^	0.880^***^	0.941^***^	0.959^***^		
Collaboration and platform	0.574^***^	0.590^***^	0.582^***^	0.760^***^	0.843^***^	0.787^***^	0.842^***^	0.854^***^	0.923^***^	
Feedback and reflection	0.666^***^	0.653^***^	0.638^***^	0.764^***^	0.849^***^	0.821^***^	0.884^***^	0.890^***^	0.986^***^	0.912^***^

^***^ indicates *p* < 0.001. Data are from the survey “Questionnaire on University PE Instructors’ Political Literacy and Its Impact in Curriculum-based Ideological and Political Education.”

First, the dimensions of political literacy demonstrated a high degree of integration. The correlation coefficients between political understanding, political identification, and political faith were extremely high (*r* = 0.952–0.988), indicating that cognition, identification, and faith constitute a closely connected and logically progressive continuum at the ideological level. In contrast, the correlations between political practice and the first three theoretical cognition dimensions, although still strong (*r* = 0.696–0.718), were significantly lower. This preliminarily confirms the finding from the descriptive analysis that translating solid theoretical cognition into effective teaching practice involves certain thresholds, potentially moderated by factors such as teaching skills and the external environment.

Second, political literacy was generally positively correlated with values development outcomes, but the strength of association varied by dimension. Overall, all dimensions of political literacy showed significant positive correlations with all dimensions of values development outcomes. Notably, political practice exhibited the strongest association with values development, particularly showing high correlations with enjoyment experience (*r* = 0.820) and character development (*r* = 0.841). This result highlights that teachers’ practical translation ability is a direct driver for enhancing course attractiveness and student moral development. In comparison, the theoretical dimensions of political literacy—understanding, identification, and faith—showed relatively weaker correlations with physical fitness (*r* = 0.587–0.591), suggesting that the promotion of students’ physical health through political literacy relies more on concrete teaching practices and behavioral guidance rather than theoretical cognition alone.

Finally, educative efficacy played a pivotal role. The analysis showed widespread and significant synergistic relationships between educative efficacy and both political literacy and values development outcomes. Among these, political practice was closely associated with all dimensions of educative efficacy, particularly self-efficacy (*r* = 0.855) and feedback and reflection (*r* = 0.764), indicating that successful teaching practices can greatly enhance teachers’ instructional confidence and promote professional reflection. Simultaneously, the near-perfect synchrony between enjoyment experience in values development and teachers’ self-efficacy (*r* = 0.931) revealed a tight bidirectional link between classroom enjoyment and teacher confidence. Furthermore, the very strong correlations within the dimensions of educative efficacy, such as between self-efficacy and collaboration and platform support (*r* = 0.923) and between self-efficacy and feedback and reflection (r = 0.986), collectively illustrate that enhancing teacher efficacy is a systematic project, dependent on the organic integration of individual beliefs, teamwork, and continuous reflection.

### 4.3 Structural equation modeling results

To test the theoretical model and hypotheses proposed in this study, a structural equation model (SEM) was constructed to analyze the pathway through which university PE instructors’ political literacy (independent variable) influences student values development outcomes (dependent variable) through educative efficacy (mediating variable) (see Table 3). The model fit indices showed: *χ*^2^/df = 2.468 (<5), NFI = 0.838, CFI = 0.896, RMSEA = 0.084. These indices indicate that the overall fit of the model is within an acceptable range, allowing for subsequent path analysis and hypothesis testing.

**Table 3:** SEM model fit indices.

Indicator	CMIN/DF	NFI	IFI	TLI	CFI	RMSEA
General criterion	<5	>0.8	>0.8	>0.8	>0.8	<0.1
Measurement model results	2.468	0.838	0.897	0.890	0.896	0.084

#### 4.3.1 Path analysis results

The standardized path coefficients and their significance levels in the model are shown in Table 4. Specifically, the path coefficient from political literacy to educative efficacy is 0.668 (*z* = 10.634, *p* < 0.001), indicating a highly significant large effect. This suggests that an improvement in teachers’ political literacy directly and effectively enhances their educative efficacy. The path coefficient from educative efficacy to values development outcomes is as high as 0.853 (*z* = 13.408, *p* < 0.001), representing an extremely strong effect. This implies that teachers’ educative efficacy is the most direct and powerful proximal factor driving the development of student values. The direct path coefficient from political literacy to values development outcomes is 0.136 (*z* = 3.409, *p* < 0.01). Although statistically significant, the effect size is small. This result preliminarily indicates that the influence of political literacy on student values is likely primarily realized through the mediating variable of educative efficacy, rather than through a direct effect.

**Table 4:** Path analysis results.

Path test			Estimate	S.E.	C.R.	*p*	STD.estimate
Educative efficacy	<---	Educative efficacy	0.493	0.046	10.634	***	0.668
Values development	<---	Educative efficacy	0.816	0.061	13.408	***	0.853
Values development	<---	Educative efficacy	0.096	0.028	3.409	***	0.136

#### 4.3.2 Mediation effect test

To precisely verify the mediating role of educative efficacy, this study employed the Bootstrap method (with 5,000 resamples) for testing. The results, as shown in Table 5, indicate that the total effect of political literacy on values development outcomes is 0.498 [95% CI (0.304, 0.689)]. Within this, the indirect effect transmitted through educative efficacy is 0.402 [95% CI (0.224, 0.621)], confirming that this mediation effect is statistically significant. However, after including the mediating variable, the direct effect of political literacy on values development outcomes becomes 0.096 [95% CI (−0.017, 0.343)]. Since its confidence interval includes zero, the direct effect is no longer statistically significant.

**Table 5:** Mediation effect analysis results.

Effect type	Path	Effects	95%CI	
			Lower	Upper
Indirect effect	Political literacy → educative efficacy → values development	0.402	0.224	0.621
Direct effect	Political literacy → values development	0.096	−0.017	0.343
Total effect	Political literacy → values development	0.498	0.304	0.689

In summary, the mediation effect test results support hypothesis H2, indicating that educative efficacy plays a substantially mediating role between political literacy and values development outcomes. This implies that teachers’ political literacy must be catalyzed by strong educative confidence and capability to effectively promote the development of student values. This finding precisely reveals the internal transmission mechanism of “literacy → belief → outcome” and clarifies that enhancing teachers’ educative efficacy is the pivotal link in achieving the goal of “Fostering Virtue through Education.”

### 4.4 Discussion

This study, through structural equation modeling and mediation effect testing, confirms that teacher educative efficacy plays a central mediating role between political literacy and the development of student values. However, this transmission pathway is not unimpeded. Based on the empirical data, we have identified a critical obstruction within it—“delayed transmission effect.” Drawing on established theoretical frameworks from educational psychology and teacher education research, this section analyzes the underlying mechanisms of these delays and explores pathways to resolve them, following the logic of “phenomenon-mechanism-solution.”

#### 4.4.1 Delay in translating political cognition into teaching practice

Descriptive analysis has already revealed a significant gap between university PE instructors’ theoretical political cognition (understanding, identification, faith) and their teaching practice capability. The mediation model in this study further indicates that political literacy must work through educative efficacy to effectively influence educational outcomes. From a theoretical perspective, this “translation delay” can be understood through the lens of the “belief-practice gap” (Fang, 1996), a well-documented phenomenon in international teacher education research wherein teachers’ espoused beliefs often fail to align with their enacted practices due to contextual constraints and insufficient pedagogical reasoning. In this study, the essence of the translation delay is an insufficient efficacy belief among instructors regarding their ability to translate internal political cognition into external teaching behaviors—a deficit that undermines the bridge between belief and practice.

This delay primarily stems from three obstacles: First, capability gap, or failure in translating concept to strategy. Even with a solid theoretical foundation, teachers generally lack the micro-level instructional design capability to translate the “political discourse system” into the “language of PE teaching” (Carl et al., 2025). For instance, while some teachers recognize the value of the “Women’s Volleyball Spirit,” they cannot deconstruct it into specific collaborative games or adversity simulation drills, leaving theory disconnected from practice. This “easier said than done” predicament directly erodes their self-efficacy, reducing their confidence in successfully implementing values education (Rutkauskaite et al., 2025). Second, lack of systemic support, misalignment of environment and incentives. The current support system fails to effectively underpin teachers’ translation practice (Prusak et al., 2024). On one hand, systematic training in ideological and political teaching tailored to the characteristics of the sports discipline is insufficient, making it difficult for teachers to break away from the path dependence of “skill delivery” (Zhou, 2024). On the other hand, the evaluation system still prioritizes quantifiable athletic achievements and skill mastery, paying insufficient attention to qualitative outcomes like the effectiveness of ideological and political education, thereby weakening the external motivation for teaching innovation (Rink, 2013). Third, ambiguous role identity, and confusion over mission and professional boundaries. Some PE instructors lack a clear perception of their role as “values educators,” still viewing ideological and political education as the exclusive responsibility of dedicated politics course teachers (Ibañez et al., 2020). This deviation in role perception reduces their initiative to actively practice political literacy at the source of motivation.

This study reveals that the accumulation of political literacy and the enhancement of self-efficacy are two sides of the same process. Profound political literacy not only provides value orientation but is itself a key source for generating self-efficacy (Jang et al., 2025). By enhancing teachers’ understanding and execution of educational policies, it directly solidifies their professional confidence. Simultaneously, the beliefs, innovative spirit, and sense of responsibility internalized through political literacy are also core traits that drive teachers’ professional development (Yuan et al., 2015).

Therefore, resolving the translation delay requires the systematic coupling of deepening political literacy with activating self-efficacy. Specific pathways include: (1) Capability Empowerment. Develop a “political concept → teaching behavior” translation toolkit (Marsh et al., 2006). Utilize thematic workshops, exemplary teaching case libraries, and embedded teaching resource packages to help teachers master strategies for translating macro narratives like “Sports Power” into lesson objectives and teaching behaviors, fundamentally boosting their confidence in instructional design and implementation. (2) Mechanism Guidance. Restructure the teaching evaluation and incentive mechanisms. Incorporate the effectiveness of values education as a core dimension of performance assessment and establish a professional development cycle of “training-practice-certification” (Liu P. et al., 2021; Liu X. et al., 2021). Use institutionalized positive feedback to drive teachers’ transition from being “knowers” to “practitioners.”

#### 4.4.2 The lag in sublimating classroom enjoyment into value internalization

Empirical data indicate that while PE courses are highly effective in stimulating students’ enjoyment, this positive emotion is not sufficiently guided and sublimated into enduring willpower and stable values. This “sublimation lag” phenomenon reveals an incomplete teaching cycle from emotional arousal to character internalization (Eloff et al., 2024). This lag can be theoretically illuminated by Self-Determination Theory (Ryan and Deci, 2000), which distinguishes between situational interest—the transient affective response triggered by engaging activities—and internalized motivation—the enduring value orientation that arises when basic psychological needs for autonomy, competence, and relatedness are satisfied. In the context of PE, classroom enjoyment represents situational interest, but its sublimation into value internalization requires deliberate pedagogical scaffolding that helps students connect their immediate experiences to broader meaning systems. Without such scaffolding, enjoyment remains ephemeral, failing to crystallize into stable character traits.

Its roots lie in dual deficiencies in teaching depth and systemic support: First, teaching practices often lack conscious “value mining.” Physical activities inherently contain setbacks and challenges, providing natural contexts for cultivating resilience and responsibility. However, if teaching objectives remain focused solely on skill acquisition and classroom engagement, without guiding students to reflect on adversities encountered in activities or recognize responsibilities within teamwork, then “enjoyment” remains a transient emotion, failing to transform into profound character traits. Instruction misses the crucial leap from “experience” to “insight.” Second, the absence of collaborative education mechanisms creates “educational silos.” PE instructors often conduct their educational practices in isolation. Questionnaire results reflect that schools fail to provide adequate cross-departmental ideological and political teaching support. Although teachers’ political literacy establishes the goal of “balancing physical development with character building,” the lack of effective collaborative platforms with ideological and political teachers and student affairs staff to co-design teaching content hinders their access to the theoretical tools and methodological support needed to translate macro goals into concrete teaching scripts.

This study confirms that collaborative platforms serve as a key external lever to break this lag. Acting as a hub for teachers to transition political literacy from “internalization” to “externalization,” they provide space for interdisciplinary dialogue and practical innovation, and are crucial for achieving resource sharing and experience transfer (Li and Hu, 2023; Yi et al., 2024). Therefore, building a systematic collaborative support network is essential: (1) *Promote institutional synergy*. Establish a multi-dimensional “PE–Ideological/PE–Student Affairs–Academic Affairs” linkage mechanism at the university level. Centered on the goal of values education, this facilitates co-research on curricula, co-organization of activities, and joint evaluation of outcomes, thereby consolidating educational efforts. (2) *Enable platform empowerment*. Foster a regular teaching research community within the institution between PE and ideological/political education teachers. Through collective lesson preparation, cross-disciplinary class observations, and similar activities, achieve methodological integration. (3) *Expand off-campus practical bases*. Allowing teachers to refine teaching materials in real-world social contexts. Furthermore, develop cloud-based resource libraries that aggregate high-quality case studies, providing teachers with sustained and accessible digital support.

#### 4.4.3 The lag in translating educative efficacy into educational outcomes

The structural equation model revealed the central mediating role of educative efficacy. Consequently, its insufficient development directly leads to inefficiency in the entire transmission chain from political literacy to final outcomes. This “efficacy-outcome lag” resonates with research on the conditions necessary for teacher efficacy to translate into student achievement (Zee and Koomen, 2016). Efficacy beliefs, while necessary, are not sufficient; they must be coupled with accurate feedback and reflective practice to generate effective instructional adaptations. In the absence of such mechanisms, even teachers with strong efficacy may fail to realize its full potential in influencing student outcomes.

Data indicate that a key bottleneck currently constraining the continuous improvement of educative efficacy lies in the weakness of feedback and reflection mechanisms. Teachers’ political literacy requires sustained external feedback and deep reflection within their teaching practice to iteratively optimize and ultimately solidify into stable teaching capability. However, the survey reveals a general lack of effective and diverse feedback channels in the current field of university PE. This makes it difficult for teachers to obtain accurate information about the effectiveness of their ideological and political teaching, thereby limiting their capacity for precise teaching reflection and improvement.

A sound feedback and reflection mechanism is the iterative driving force that promotes the internalization of teachers’ political literacy and ensures the continuous optimization of the work of “Fostering Virtue through Education” (Gormley-Heenan and McCartan, 2009; Oh et al., 2025). On one hand, it facilitates the transformation of teachers’ theoretical knowledge, national policies, and personal beliefs into conscious action. On the other hand, it provides precise navigation for teaching improvement through diverse feedback sources. To activate this iterative drive, it is necessary to construct a closed-loop system that promotes professional growth: first, establish a multi-source feedback mechanism. Build a three-tier feedback system integrating “immediate student feedback, diagnostic peer evaluation, and expert supervisory assessment.” This helps teachers comprehensively and accurately identify their strengths and weaknesses in values education. Second, deepen reflective practice. Integrate systematic political learning with concrete teaching reflection. Guide teachers not only to study educational policies and Core Socialist Values but also, using these as guidance, to repeatedly examine their own teaching cases and student feedback. Through this process, they can continuously enhance their subjective consciousness of “cultivating virtue through sports” and their professional confidence, completing the transition from “experienced teacher” to “reflective practitioner.”

## 5 Conclusion and implications

Through empirical investigation and model testing, this study systematically examined the mechanism through which university PE instructors’ political literacy influences the development of student values. The core conclusion is that teachers’ political literacy is not a static reservoir of theoretical knowledge, but rather a dynamic, composite professional competency comprising political understanding, political identification, political faith, and political practice. A key finding of the research is the revelation that educative efficacy plays a substantially mediating role in this influence mechanism, and the identification of three delayed transmission effects within the pathway: the delay in translating political cognition into teaching behaviors, the delay in sublimating classroom enjoyment into value internalization, and the delay in converting educative efficacy into educational outcomes.

Further analysis of the underlying mechanism indicates that the cultivation and development of teachers’ political literacy relies on a synergistic system: its accumulation stems from the endogenous drive of self-efficacy, its deepening depends on the external support provided by collaborative platforms, and its continuous optimization is sustained by the iterative momentum generated by feedback mechanisms.

Based on these findings, this study constructs a systematic enhancement path integrating a trinity of “stimulating endogenous motivation-optimizing the external environment-ensuring developmental mechanisms.” This path practically indicates: activating teachers’ endogenous motivation through capacity building and institutional incentives; optimizing the teaching support environment through cross-departmental collaboration and platform development; and ensuring the continuous development of their professional capabilities through diverse feedback and reflective practice.

Although this study is conducted within the specific policy context of China’s “Curriculum-based Ideological and Political Education,” the influence mechanism of “political literacy → educative efficacy → values cultivation” among university PE instructors, as revealed in the study, holds universal significance that transcends specific cultural or political contexts. Today, as countries around the world increasingly emphasize the integration of core values or civic education into school curricula, how to enable subject teachers to become effective values educators is a common challenge. This study finds that it is insufficient for teachers merely to possess correct values or policy knowledge (political literacy). The key lies in whether they believe they have the ability to effectively transmit these values to students (educative efficacy). This sense of efficacy serves as the intrinsic motivation for inspiring teaching innovation, sustaining commitment, and addressing challenges among teachers. Therefore, any country or educational system that aims to conduct values education through subject teaching should focus on cultivating and enhancing teachers’ “educative efficacy” through professional development programs, collaborative culture building, and supportive evaluation mechanisms.

## Data Availability

The raw data supporting the conclusions of this article will be made available by the authors, without undue reservation.
